# Feasibility study of strengthening the public–private partnership for tuberculosis case detection in Bandung City, Indonesia

**DOI:** 10.1186/s13104-017-2701-y

**Published:** 2017-08-14

**Authors:** Bony Wiem Lestari, Nita Arisanti, Adiatma Y. M. Siregar, Estro Dariatno Sihaloho, Gelar Budiman, Philip C. Hill, Bachti Alisjahbana, Susan McAllister

**Affiliations:** 1 TB-HIV Research Centre, Faculty of Medicine, Universitas Padjadjaran, Bandung, Indonesia; 2 Department of Public Health, Faculty of Medicine, Universitas Padjadjaran, Bandung, Indonesia; 3 Centre for Economics and Development Studies, Department of Economics, Faculty of Economics and Business, Universitas Padjadjaran, Bandung, Indonesia; 4 Faculty of Electrical Engineering, Telkom University, Bandung, Indonesia; 5 Centre for International Health, Department of Preventive and Social Medicine, University of Otago, Dunedin, 9054 New Zealand

**Keywords:** Private practitioner, Tuberculosis, Mobile phone application

## Abstract

**Objective:**

Private practitioner’s (PPs) collaboration for detection, diagnosis and treatment of tuberculosis (TB) is recommended by the World Health Organization and encouraged by the Indonesian National TB control programme. TB case management by PPs, however, are mostly not in line with current guidelines. Therefore, we developed an intervention package for PPs comprising of TB training, implementation of a mobile phone application for notification of TB cases and a 6-month regular follow-up with PPs. This study aimed to evaluate the feasibility of the intervention package to increase TB case detection and notification rates among PPs in five community health centre areas in Bandung City, Indonesia.

**Results:**

A total of 87 PPs were registered within the study area of whom 17 attended the training and 12 had the mobile phone application successfully installed. The remaining five PPs had phones that did not support the application. During the follow-up period, five PPs registered patients with TB symptoms and cases into the application. A total of 36 patients with TB symptoms were identified and 17 were confirmed TB positive.

## Introduction

In 1991, the directly observed treatment strategy (DOTS) was instigated for provision of tuberculosis (TB) treatment services. Yet despite this, and other concerted efforts to control TB, each year more than nine million new cases of TB are diagnosed globally [1]. Moreover, the incidence rate is declining only by 2% per year, therefore, considerable scale-up of effort is considered necessary for the elimination target, of less than 1 case/1,000,000 population by 2050 [2]. The World Health Organization (WHO) [3] has recommended involving the private health sector, known as Public–Private Mix (PPM), as an essential part of scale-up. This is particularly important in low- and middle-income (LMIC) countries where up to 50% of people reportedly first seek care from a private practitioner (PP), and where the level of care is often sub-optimal [4–8].

Indonesia, with an estimated prevalence of 647 per 100,000 population, is ranked the second highest TB-burden country in the world [1]. The National TB control programme (NTP) has an established system of diagnosing and treating TB through community health centres (CHCs), public hospitals, and laboratories. Engagement of PPs for TB care, while currently not undertaken, is among the NTP’s priority goals. Previous studies have recognized barriers to effective PP engagement including a lack of incentive, complicated TB reporting and referral systems, and lack of communication and trust between the private and public sectors [9, 10]. Our feasibility study in allocated districts of Bandung City sought to investigate and understand the barriers to involving PPs in TB control, develop a system to reduce those barriers, and evaluate the feasibility of this system to increase the number of correctly diagnosed and notified new TB cases.

## Main text

### Methods

#### Research setting and study overview

Bandung city, Indonesia, has an estimated population of 2.5 million people. Seventy-three CHCs provide the population with general health services, including TB management. After consultation with the City Health Office, five CHC areas were selected for the study and an initial needs-assessment carried out to identify important issues in TB management. An intervention was designed and implemented for a 6-month follow-up period from October 2015 to March 2016. Ethical approval was obtained from the Universitas Padjadjaran Ethics Committee (166/UN6.C1.3.2/KEPK/PN/2015).

#### Needs assessment

The needs assessment comprised the following:

1. *Meetings with key stakeholders* Three meetings were held with NTP staff to discuss possible intervention/s to strengthen PPM.
2. *In*-*depth interviews* One TB nurse; two CHC in-charge; one TB programme coordinator, and two PPs were selected purposively for interview about current practice, difficulties in diagnosis and reporting of TB, and their perception of existing PPM collaboration. Interviews were recorded, then transcribed and manually coded by the principal investigator. Themes were identified and reviewed by the research team.
3. *Baseline questionnaire with PPs* Lists of all PPs registered with the relevant CHC were obtained. A pre-tested questionnaire about the clinic, practitioner qualifications, and their experience with TB patients was prepared. Two trained medical doctors contacted each PP by phone to arrange a visit for interview.

#### Interventions

Three main interventions were developed to address the identified barriers:

1. A referral and report-back system using a mobile phone application was developed in collaboration with information technology (IT) programmers from Telkom University, Bandung. The information included was a simplified version of NTP forms: patient’s identity, symptoms, diagnostic examinations, TB type, treatment, and follow-up care. Inputted information was sent to a secure centralised server and could be monitored by the research team in a web-based application. An account and password was created for each eligible user.
2. A 1-day training on TB was held for PPs including:
  - Case management.
  - Reporting and recording.
  - Introducing the mobile phone application.

Participants were given written information about DOTS, the National TB guidelines, and a certificate of proficiency issued by the Indonesian Medical Association that provided PPs with credits that could be used as proof of continuing medical education for renewing their licence to practice.

3. Regular follow-up was undertaken with each PP over 6 months. A research assistant first visited the PP to provide the login account, instructions, and an application operations manual, then regularly phoned or visited to ask about any TB suspects or cases and to remind them to input data to the application.

#### Evaluation

The main outcomes of interest and evaluation methods were:

- The number of new presumptive TB cases seen, and cases diagnosed by PPs.
- The acceptability and use of the application. A structured questionnaire was completed with each PP after 6 months to explore their experience of using the application and suggestions for improvement.
- The costs of the intervention and maintenance were calculated using a micro-costing approach [11] and were constructed from recurrent and capital costs using the health system perspective [12]. Costs borne by patients were excluded. Recurrent costs consisted of personnel cost, supplies, and other services (interview translation, 12-month software maintenance). Capital costs included training of PPs and development of the web and client applications. Total costs were divided by the number of people with TB symptoms, TB cases diagnosed, and number of PPs. Costs were measured in US dollars, converted from Indonesian Rupiah (2015 exchange rate [13]).

## Results

### Needs assessment

#### In-depth interviews

The following key themes were identified:

1. The lack of TB drugs and diagnostic facilities in private clinics required practitioners to refer patients, but patients often refused because of the greater effort required, long waiting times, and lack of trust in the public system. For the PP, referring a patient effectively meant that patients were then ‘lost’ as a client.
2. TB reporting is complicated, takes a long time and there is a lack of supervision from CHCs.

#### Baseline interviews with private practitioners

A total of 87 PPs and 12 private clinics were registered to practice in the study area of whom 27 (31%) were interviewed and completed the questionnaire (65% female; average age—38 years (range 23–80) (Table 1). Of the 60 PPs not included, the main reasons were: unavailable for interview after three contact attempts (n = 41); no longer practicing (n = 13); working in beauty therapy only (n = 4); unrecognisable address (n = 2).

**Table 1:** Information from baseline questionnaire with Private Practitioners (n = 27)

Characteristics	
Solo practice	48%
Average length of time practicing in this clinic/location	10 years
Practicing medicine in another location	74%
Previously participated in a PPM project	7%
Monthly report of patients sent to CHC	63%
Follow-up contact with CHC staff in past year	22%
Average length of time for patient to get from PP clinic to CHC	12 min
Average number of patients with TB symptoms seen in the past year (range)	11 (0–150)
Average number of TB cases diagnosed in the past year (range)	6 (0–54)
Normal place of referral for sputum smear for patients with TB symptoms	
CHC	41%
Laboratory (private or city health office laboratory)	26%
Hospital	7%
Other (public/government lung centre)	26%
Normal place of treatment for positive TB cases	
Own clinic	21%
CHC	42%
Hospital	8%
Others (public/government lung centre)	29%
Correct answer given to the question: “Which TB signs and symptoms are the most important for investigation of TB?”	59%

*PPM* public–private mix, *PP* private practitioner, *CHC* community health centre, *TB* tuberculosis

### Intervention and follow-up

**Fig. 1:**
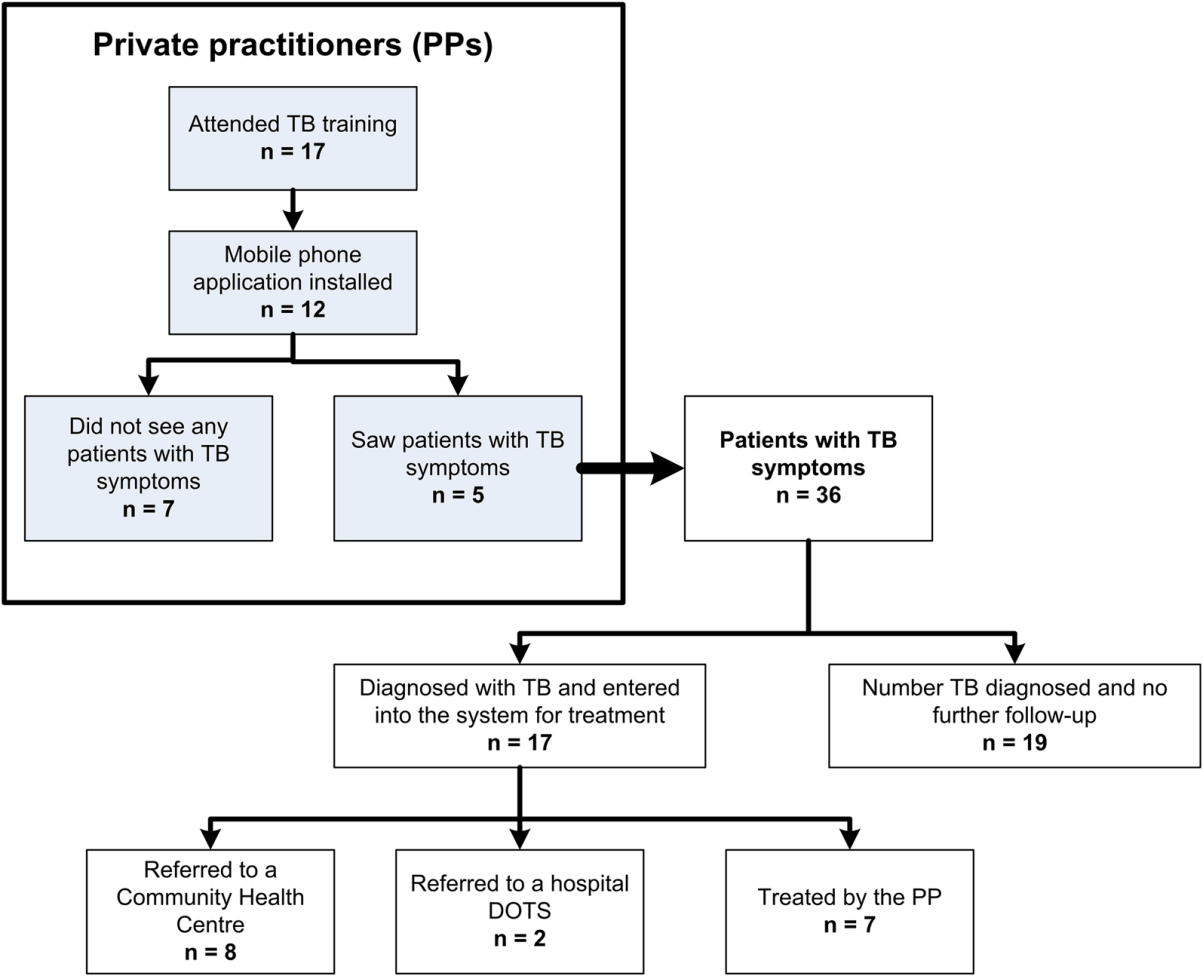
Flow chart of private practitioner (PP) participation, detection and referral of patients with tuberculosis (TB) symptoms

A total of 17 of the 27 PPs participated in the training. The mobile phone application was successfully installed for 12 PPs. The remaining five had phones that did not support the application. During the follow-up period, five PPs registered 36 patients with TB symptoms into the application, 17 of whom were diagnosed with TB and entered into the system for treatment follow-up; seven by the PP, eight to CHC, and two to hospital DOTS. The remaining 19 patients were not diagnosed with TB and there was no further follow-up (Fig. 1).

### Acceptability

All PPs who attended the training provided informal feedback on its usefulness. Of the five PPs who saw symptomatic TB patients, all agreed that the application was useful for recording and reporting. Some key issues identified included: the need for a strong internet connection; an additional reminder system to alert the PP if the patient does not complete any treatment follow-up; an individualised patient list for each PP. Fear of inspection by authorities if they manage TB cases incorrectly was also an expressed concern.

### Cost analysis

The total cost of setting up, and 1-year maintenance of the PPM system was $4166.29 with the largest proportion spent on training (34%) followed by development of the application (31%). Maintenance costs were $448 per year ($37 per month). The unit cost for each TB report (patients with TB symptoms) was $115.73, $245.08 for each confirmed positive TB case, and $347.19 per PP who had the application installed (n = 12) (Table 2).

**Table 2:** Costs of developing the public–private-mix system (US$)

Item	Value per item	Total
Capital costs		
Web and client application	1288.52 (30.9%)	
Training	1422.81 (34.2%)	
Sub total		2711.33
Recurrent costs		
Personnel	645.29 (15.5%)	
Supplies	159.90 (3.8%)	
Other activities (application maintenance)	649.77 (15.6%)	
Sub total		1454.96
Total cost		4166.29 (100%)
Cost per TB positive case		245.08
Cost per TB report		115.73
Cost per private practitioner		347.19

Using Indonesian Rupiah 13,389.41/US$

## Discussion

In countries where approximately 50% of individuals first seek care from a private health practitioner, the importance of engaging PPs in TB case detection is well recognised [4, 5, 14]. We have shown that, with adequate training and support, a mobile phone application for reporting TB-symptomatic individuals and diagnosed TB cases was acceptable and utilised by PPs, albeit in a small number of PPs. With further refinement and trialling, it has the potential to increase TB notification rates.

Despite PPs in Indonesia having to officially register with their local CHC, these lists were not well maintained. Once PPs were identified, we found that many worked in multiple locations and were too busy to commit to undertaking extra work. As a result, only 17 PPs (out of a possible 87 registered) participated in our training and it is likely that they were already more aware about TB and willing to collaborate with the NTP. In Pakistan, 50% of practitioners participated in a PPM training but had limited subsequent involvement despite receiving frequent supervision [15]. In South India only 33% (46 out of 138) of PPs had ever notified TB cases with the main reasons being a lack of time, issues related to patient confidentiality, and fear of offending patients [16]. Even after formalising and legislating for mandatory TB notification in India there continued to be difficulty in engaging PPs [17, 18]. In our study, only a small number of PPs saw the majority of TB patients. The main reasons for this appeared to be longer clinic opening hours, more convenient location, and that these clinics were known to have more experience in TB diagnosis. Given the limited resources in most LMIC, and the time constraints on many PPs, a focus on supporting these key PPs who treat the most patients may be the best use of resources [14].

It was of concern that only 59% of the PPs interviewed were able to answer about TB symptoms correctly. The fact that many PPs don’t have good knowledge about TB case management has also been documented in other settings [19–21]. The limited training on TB in the medical curriculum and limited opportunities for continuing medical education are likely contributing factors [7]. Finding appropriate ways to increase knowledge about TB therefore needs to be included in any project with PPs [9].

The cumbersome nature of paper reporting, multiple forms required, and lack of any supervision were some of the barriers to case notification identified in our study. While our mobile-phone application was found to be acceptable to the PPs who used it, more work is required to ‘fine-tune’ the application. The fact that only 12 out of 17 PPs successfully installed the application, a standardised mobile phone could be provided to PPs at a relatively modest cost to increase participation. With the growing accessibility of the internet and mobile electronic devices in LMIC, introducing mobile phone use into public health is not only a tool for data capture but also has the potential of changing communication between health practitioners and the provision of health services [18, 22, 26]. Implementation of an effective electronic reporting system for TB in Indonesia would require investment for its initial establishment, close monitoring and supervision—particularly in the early stages after establishment—and a strong commitment from the NTP.

The costs involved in establishing the PPM system in our study compared favourably with those elsewhere. The before-diagnosis cost of engaging PPs in a study in Jogjakarta in 2004 [23], after adjusting for inflation to 2015 [24], was US$285.22 per TB report and US$461.44 per confirmed TB case, which was 59 and 47% higher than the cost per unit in our study which is most likely due to the use of the mobile application in our study.

## Conclusions

Further development and refinement of the training and mobile phone application, and an intervention trial in a larger population would enable each step we have initiated in this study to be formally evaluated for the effectiveness in increasing TB notifications.

## Limitations

This was a feasibility study in a large urban city in Indonesia. As such, it was not intended to show effectiveness at increasing TB notification rate but rather provided a means for establishing and engaging community partnerships, developing a system to reduce barriers to TB control amongst PPs, and understanding whether that system was acceptable for use by key stakeholders [25]. The small number of PPs who participated in the training and subsequent use of the mobile phone application is an indication of the further work required before such a project could be truly effective.

## Abbreviations

CHC: community health centre
DOTS: directly observed treatment strategy
IT: information technology
LMIC: low and middle-income countries
NTP: National TB control programme
PP: private practitioner
PPM: public–private mix
TB: tuberculosis
WHO: World Health Organization

## References

[1] World Health Organization. Global tuberculosis report. 20th ed. NLM classification WF 300. Geneva; 2015.

[2] World Health Organization. Global tuberculosis control: WHO report 2011. WHO/HTM/TB/2011.16. Geneva; 2011.

[3] World Health Organization. Stop TB policy paper: contributing to health system strengthening: guiding principles for national tuberculosis programmes. WHO/HTM/TB/2008.400. Geneva; 2008. 24921118

[4] Uplekar M, Pathania V, Raviglione M (2001). Private practitioners and public health: weak links in tuberculosis control. Lancet.

[5] Lönnroth K, Castro KG, Chakaya JM, Chauhan LS, Floyd K, Glaziou P (2010). Tuberculosis control and elimination 2010–50: cure, care, and social development. Lancet.

[6] Satyanarayana S, Nair SA, Chadha SS, Shivashankar R, Sharma G, Yadav S (2011). From where are tuberculosis patients accessing treatment in India? Results from a cross-sectional community based survey of 30 districts. PLoS ONE.

[7] Mahendradhata Y, Lestari T, Probandari A, Indriarini LE, Burhan E, Mustikawati D (2015). How do private general practitioners manage tuberculosis cases? A survey in eight cities in Indonesia. BMC Res Notes.

[8] Wells WA, Ge CF, Patel N, Oh T, Gardiner E, Kimerling ME (2011). Size and usage patterns of private TB drug markets in the high burden countries. PLoS ONE.

[9] Putra IWGAE, Utami NWA, Suarjana IK, Duana IMK, Astiti CID, Putra I (2013). Factors associated to referral of tuberculosis suspects by private practitioners to community health centres in Bali Province, Indonesia. BMC Health Serv Res.

[10] Probandari A, Utarini A, Lindholm L, Hurtig A-K (2011). Life of a partnership: the process of collaboration between the National Tuberculosis Program and the hospitals in Yogyakarta, Indonesia. Soc Sci Med.

[11] Drummond MF, Sculpher MJ, Claxton K, Stoddart GL, Torrance GW (2015). Methods for the economic evaluation of health care programmes.

[12] Afriandi I, Siregar AY, Meheus F, Hidayat T, van der Ven A, van Crevel R (2010). Costs of hospital-based methadone maintenance treatment in HIV/AIDS control among injecting drug users in Indonesia. Health Policy.

[13] The World Bank. Official exchange rate (LCU per US$, period average). http://data.worldbank.org/indicator/PA.NUS.FCRF (2015). Accessed 17 Jan 2017.

[14] World Health Organization and Stop TB Partnership. Ninth meeting of the subgroup on public-private mix for TB care and control and global workshop on engaging large hospitals. http://www.who.int/tb/careproviders/ppm/NinthPPMreport.pdf?ua=1 (2013). Accessed 9 May 2016.

[15] Pethani A, Zafar M, Khan AA, Rabbani Sana U, Ahmed S, Fatmi Z (2015). Engaging general practitioners in public–private mix tuberculosis DOTS program in an urban area in Pakistan: need for context-specific approach. Asia Pac J Public Health.

[16] Thomas BE, Velayutham B, Thiruvengadam K, Nair D, Barman SB, Jayabal L (2016). Perceptions of private medical practitioners on tuberculosis notification: a study from Chennai, South India. PLoS ONE.

[17] Lal S, Sahu S, Wares F, Lönnroth K, Chauhan L, Uplekar M (2011). Intensified scale-up of public–private mix: a systems approach to tuberculosis care and control in India. Int J Tuberc Lung Dis.

[18] Nagaraja S, Achanta S, Kumar A, Satyanarayana S (2014). Extending tuberculosis notification to the private sector in India: programmatic challenges?. Int J Tuberc Lung Dis.

[19] Al-Maniri AA, Al-Rawas OA, Al-Ajmi F, De Costa A, Eriksson B, Diwan VK (2008). Tuberculosis suspicion and knowledge among private and public general practitioners: questionnaire based study in Oman. BMC Public Health.

[20] Vandan N, Ali M, Prasad R, Kuroiwa C (2009). Assessment of doctors’ knowledge regarding tuberculosis management in Lucknow, India: a public–private sector comparison. Public Health.

[21] Yimer SA, Holm-Hansen C, Bjune G (2011). Assessment of knowledge and practice of private practitioners regarding tuberculosis control in Ethiopia. J Infect Dev Ctries.

[22] Fraser H, Biondich P, Moodley D, Choi S, Mamlin B, Szolovits P (2005). Implementing electronic medical record systems in developing countries. Inform Prim Care.

[23] Mahendradhata Y, Probandari A, Ahmad RA, Utarini A, Trisnantoro L, Lindholm L (2010). The incremental cost-effectiveness of engaging private practitioners to refer tuberculosis suspects to DOTS services in Yogjakarta, Indonesia. Am J Trop Med Hyg.

[24] The World Bank. Inflation consumer prices (annual %). http://databank.worldbank.org/data/reports.aspx?Code=FP.CPI.TOTL.ZG&id=af3ce82b&report_name=Popular_indicators&populartype=series&ispopular=y# (2016). Accessed 17 Jan 2017.

[25] Bowen DJ, Kreuter M, Spring B, Cofta-Woerpel L, Linnan L, Weiner D (2009). How we design feasibility studies. Am J Prev Med.

[26] Khan AJ, Khowaja S, Khan FS, Qazi F, Lotia I, Habib A (2012). Engaging the private sector to increase tuberculosis case detection: an impact evaluation study. Lancet Infect Dis.

